# Reducing Phosphorus Loss in Short-Cycle Horticultural Production Using Activated Aluminum-Amended Substrates and Modified Fertigation Practices

**DOI:** 10.3390/plants13172473

**Published:** 2024-09-04

**Authors:** Damon Edward Abdi, Jeffrey Beasley, Jeb Fields

**Affiliations:** 1 Hammond Research Station, Louisiana State University Agricultural Center, 21549 Old Covington Hwy, Hammond, LA 70403, USA; jfields@agcenter.lsu.edu; 2 Department of Biology, University of North Carolina Pembroke, 1403 Old Main Rd., Pembroke, NC 28372, USA; jeffrey.beasley@uncp.edu

**Keywords:** activated aluminum, marigolds, phosphorus, nutrient leaching, adsorption

## Abstract

To support growth, short-cycle horticultural crops require readily available nutrients. However, this often leads to nutrient leaching. Implementing best management practices in production decisions like incorporating fertilizer retaining amendments to substrates or modifying fertilization programs can mitigate nutrient losses to the environment and associated costs. This study examined using an activated aluminum (AA) material as a substrate amendment to retain phosphorus (P) within containers while also assessing methods to reduce P fertilization in *Tagetes* production over a six-week production cycle. A commercial peat moss substrate (PL) pre-loaded with nutrients was amended with AA, enabling comparisons between substrates with and without AA. Enhanced fertilizer practices involved supplementing the initial nutrients by applying a weekly fertigation solution including nitrogen and potassium over the six weeks, but P for either 0, 2, 4, or 6 weeks. The incorporation of AA significantly reduced P leaching losses by 89.5–97.7%, compared to the PL substrates receiving P the entire six weeks. Regardless of substrate or fertilizer management, all *Tagetes* had equivalent sizes (growth index) and aboveground biomass. The results indicate that amending substrates with AA and/or reducing additional P inputs are effective strategies to minimize P leaching without compromising *Tagetes* quality.

## 1. Introduction

Floricultural production is a major agricultural sector accounting for >6.5 billion USD in total sales in 2022 [1]. Producing floriculture crops typically involves a short-term production cycle, and crops are almost always grown in soilless substrates. Soilless substrates alone usually do not contain sufficient concentrations of mineral nutrients to sustain crop cycles; thus, external application of immediately available nutrients is often necessary to produce marketable crops. This often manifests in applying water-soluble fertilizers (WSF) via drenching or fertigation to provide these essential elements, rather than controlled release fertilizers which may not release sufficient nutrients within the brief production cycle. Fertilizers are often applied in excess of plant needs to ensure that nutrient limitations do not inhibit growth, known as the ‘Sprengel–Liebig law of the minimum’ as described by Epstein and Bloom [2], Lea-Cox and Ristvey [3], and Owen et al. [4]. Soilless substrates are engineered to provide ideal conditions for plant growth, with facilitating ample drainage a primary consideration; however, this is often accompanied by frequent irrigation practices to maintain substrate hydration. The combination of high rates of immediately available nutrients and the frequent irrigation endemic to floricultural crops lends themselves to economic and environmental concerns regarding fertilizer leaching and loss downstream.

With magnified attention on the sustainability of horticultural production from both legislators, consumers, and the public at large, efforts to mitigate fertilizer loss may encompass a spectrum of best management practices (BMPs). Replacing readily soluble fertilizer forms with slower-releasing nutrient sources can be effective for horticultural crops grown under longer production cycles [5,6,7,8]; however, this may not be suitable for floriculture crops with shorter production times. Modifying fertilizer timing may be effective, particularly when using controlled release fertilizers (CRFs) in nursery crops, where Yeager et al. [9] suggested nursery producers split CRF applications over several events in lieu of a single, large fertilizer application. Majsztrik et al. [10] noted that growers often apply fertilizer rates based on container size rather than the taxa in production, a consideration which may be cumbersome to change given the expansive diversity of taxa that are grown and limited knowledge of species-specific nutritional needs, particularly with herbaceous plants [11]. While a more prescriptive approach to a nutrient management plan can reduce the downstream effects of fertilizer loss to the environment, growers may prefer less involved practices that could be uniformly applied to a wider range of crops.

With nearly all floriculture crops produced in soilless substrate, modifying container substrates to improve nutrient retention may be a practice that can be ubiquitously employed across taxa. Nitrogen (N) and phosphorus (P) are essential elements for the growth and development of plants in horticultural production; however, these nutrients spur the growth of harmful algal blooms observed globally when transported via runoff water to receiving waterbodies. While both nutrients are culpable for the algal blooms, it is often P that is the limiting factor (particularly in freshwater ecosystems). Furthermore, the proclivity of N to undergo transformations into different states (e.g., denitrification to gaseous forms) represents biologically mediated pathways towards removal of this element from runoff water. This trait is not shared with P, as P removal methods typically employ sequestration mechanisms (i.e., adsorption to charged surfaces or biological uptake). As P often exists in substrates as an anion, enhancing the number of exchange sites for anionic elements in a substrate represents a pathway to retaining P within the growing medium and reducing loss to the environment.

Peat moss is typically a primary component in floricultural substrates, with cation exchange capacities (CECs) varying between peat species and sources [12]. Substrate components with higher CECs may not necessarily provide sufficient anion retention; however, efforts to identify substrate components that can sorb negatively charged nutrients have been observed in several materials of interest. Aggregates such as palygorskite-bentonite industrial minerals [13], calcined clays [14], and zeolite [15] have been shown to reduce P leaching. Shreckhise and Altland [16] utilized ferrous sulfate-amended pine bark as a base layer in nursery containers to adsorb P, reducing P loss by 22–73% compared to non-amended pine bark substrates. Blending anionic-adsorbent materials into the entirety of a substrate has also yielded reductions in P leaching; Abdi et al. [17] investigated three rates of an activated aluminum (AA) material to reduce P leaching in the production of *Tagetes patula*, yielding substantial reductions in P leaching loss without sacrificing crop quality.

Activated aluminum is a granular material that possesses properties such as high porosity and anion affinity [18,19,20,21], making it suitable for a range of uses including water filtration [22,23,24] and horticulture applications, including a patent to use P-rich AA as a slow-release source of P [25,26]. Despite concerns regarding Al phytotoxicity and leaching potential [27,28,29,30,31], Abdi et al. [17] observed minimal leaching of Al and no negative effects on the growth of *Tagetes*. This may be attributed to the pH of substrate being maintained within a suitable range for both plant growth [32] and AA stability [33]. A primary observation from the 2023 study by Abdi et al. [17] was that even at the lowest rate of AA blended into the substrate, P leaching losses were reduced by 69–87%. Additionally, the leaching of several other elements was reduced, and Al leaching was insignificant.

This study expands upon the use of AA as a soilless substrate amendment to reduce anionic element loss by incorporating another BMP—modifying fertilizer practice. In this study, only one rate of AA aluminum was blended into a commercially available, nutrient-charged peat-based floriculture mix (PL) for the growth of *Tagetes* over a six-week period and compared with a control of the PL mix without AA. Fertilizer was supplied weekly by individually blending water-soluble forms of N, P, and K into one solution. While additional N and K were supplied for every fertigation event, supplemental P was either never supplied or applied for a 2-, 4-, or full 6-week period. Nutrient leachate was collected weekly, with the total load of investigated elements quantified. The growth and visual quality of *Tagetes* was similarly assessed. It was hypothesized that AA would maintain its value as a soilless substrate component with regards to reducing P loss in leachate, and that reduced P applications may not manifest in reduced crop growth, as more P may be retained within-container and/or initial levels of P in the PL mix may be sufficient for a short-cycle crop.

## 2. Results

### 2.1. Plant Growth and Biomass

**Figure 1:**
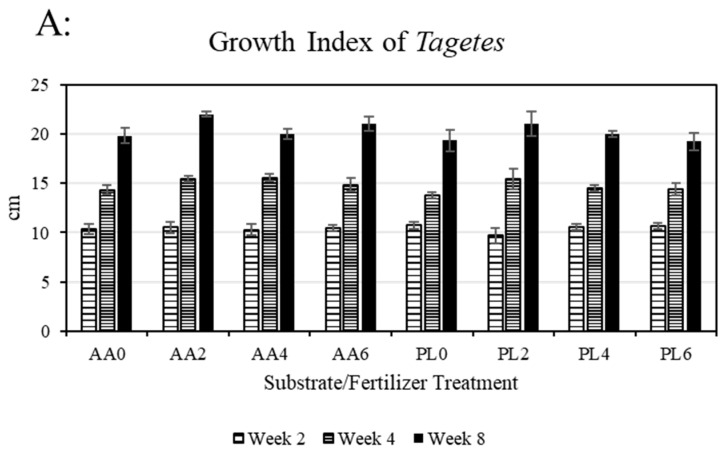

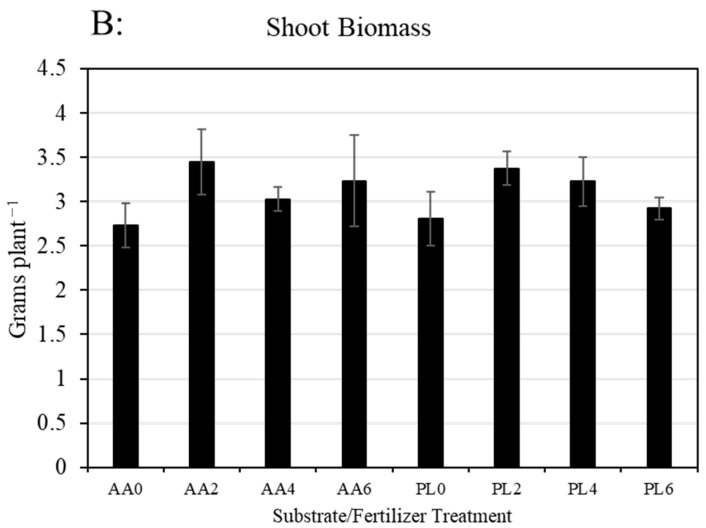
(**A**) Growth index of *Tagetes* at three stages throughout the study (mean and standard error displayed; there were no differences between treatments when analyzed using Tukey’s honestly significant difference at α = 0.05); (**B**) shoot biomass at the conclusion of this study (mean and standard error displayed; there were no differences between treatments when analyzed using Tukey’s honestly significant difference at α = 0.05). Treatments include substrate (PL = unamended peat substrate; AA = activated aluminum-amended peat substrate) and weeks receiving additional phosphorus fertilizer (0, 2, 4, or 6 weeks).

Growth index, a general assessment of above-ground plant volume, was equivalent (*p* > 0.05) for all treatments on each of the three sample dates (Figure 1A). This culminated in an average final growth index of 20.3 cm (±0.3 standard error) across all substrate and fertilizer treatments. In assessing the effects of substrate and fertilizer treatment, there were no main effects or interactive effects on any of the three sample dates (week 2: substrate *p* = 0.9538; fertilizer treatment *p* = 0.799; interaction *p* = 0.5918; week 4: substrate *p* = 0.2078, fertilizer treatment *p* = 0.1206, interaction *p* = 0.8179; week 6: substrate *p* = 0.167, fertilizer treatment *p* = 0.1233, interaction *p* = 0.7344). Similarly, shoot biomass was equivalent between all treatment combinations (Figure 1B), and there were no significant main effects regarding substrate type (*p* = 0.8991) or fertilizer treatment (*p* = 0.2231), nor interaction (*p* = 0.8435). Final shoot biomass averaged 3.1 g dry weight (±0.10) across all treatment combinations.

### 2.2. Container Leachate

Leachate was assessed weekly for a total of six weeks. Through measurement of the volume of leachate collected and analysis of a subsample to assess the concentration of the ions of interest, the total load leached from containers was calculated. Weekly loss from container substrates as well as total mass collected for each ion throughout the entirety of the study was quantified.

#### 2.2.1. Leachate Volume

The volume of leachate was equivalent in all treatments for each week of the study; however, the volume of water varied between weeks: week 1 (average volume of all treatments was 0.79 L ± 0.017, week 2 (0.45 ± 0.01), week 3 (0.20 ± 0.02), week 4 (1.65 ± 0.001), week 5 (0.43 ± 0.02), and week 6 (1.36 ± 0.03). The universal increases in leachate volume observed in weeks 4 and 6 were in response to increased irrigation demands, which were applied uniformly to all treatments.

#### 2.2.2. pH and Electrical Conductivity

**Figure 2:**
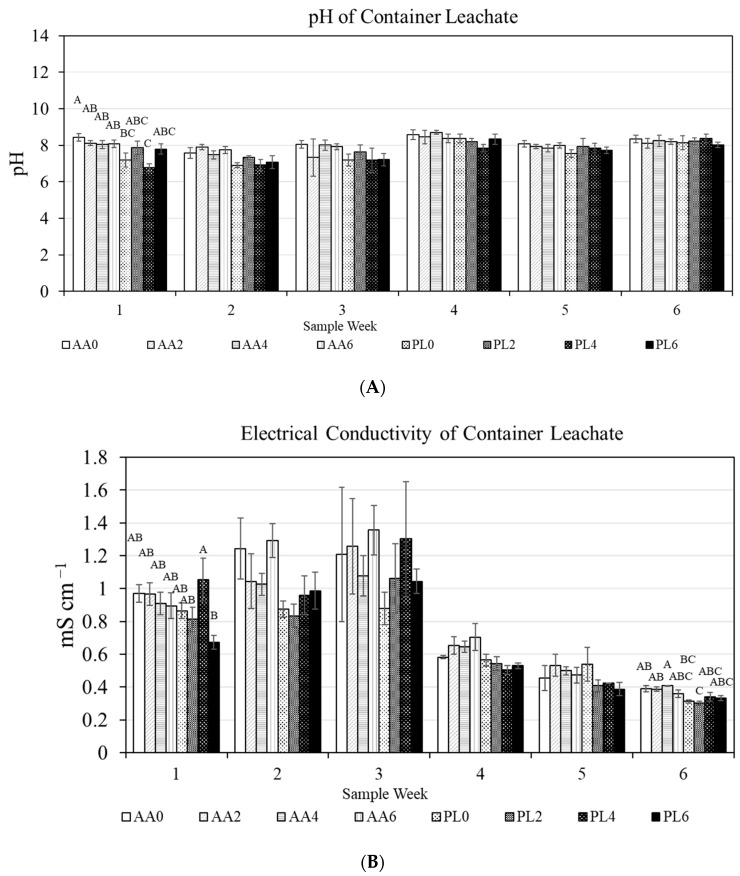
(**A**) pH of container leachate throughout the study (mean and standard error displayed; Tukey’s honestly significant differences at α = 0.05 on a given date are represented by varying letters). (**B**) Electrical conductivity (EC) of container leachate throughout the study (mean and standard error displayed; Tukey’s honestly significant difference at α = 0.05 on a given date is represented by varying letters). Treatments include substrate (PL = unamended peat substrate; AA = activated aluminum-amended peat substrate) and weeks receiving phosphorus fertilizer (0, 2, 4, or 6 weeks).

Leachate pH was generally equivalent between treatments (Figure 2A), with the exception being week 1, when AA0 had a greater pH than PL4, with all other treatment combinations being equivalent to each other. From weeks 2–5, there were no differences in pH between treatments. In assessing weekly pH, there was a substrate main effect over the first two weeks (week 1: *p* = 0.0004; week 2: *p* = 0.0013), and no fertilizer main effects nor substrate/fertilizer interactive effects on any day (*p* > 0.05). The overall pH was stable throughout the entirety of the study, where the average pH was 8.17 (±0.11) for AA0, which was greater than both PL0 (7.56 ± 0.15) and PL4 (7.49 ± 0.18). The average pH throughout the entirety of the study for the other treatments were all universally equivalent (AA2: 7.97 ± 0.18; AA4: 8.05 ± 0.12; AA6: 8.05 ± 0.08; PL2: 7.86 ± 0.13; and PL6: 7.69 ±0.13). A main substrate effect was observed for the study-long average pH (*p* < 0.0001); however, no fertilizer practice main effects (*p* = 0.7649) or interactive effects (*p* = 0.2449) were observed.

Leachate electrical conductivity (EC) provides a non-speciated assessment of fertilizer content in container leachate. In general, EC was equivalent between treatments on most sample dates (including every week from weeks 2–5), but differences were observed in weeks 1 and 6 (Figure 2B). In week 1, PL4 had a greater EC than PL6, with all other treatments equivalent to each other. Conversely, in week 6, AA4 had a greater leachate EC than either PL0 or PL2. In assessing weekly EC, there were significant substrate main effects in weeks 2 (*p* = 0.0089), 4 (*p* = 0.0012), and 6 (*p* < 0.0001), no fertilizer practice main effects on any date (*p* > 0.05), nor any interactive effects on any date (*p* > 0.05).

#### 2.2.3. Phosphorus Leachate

The concentration of phosphorus differed between treatments on every sample date, with significant main effects from substrates on each of the six sample weeks (*p* < 0.0001 for all weeks), P fertilizer practices on four sample weeks (week 1 *p* = 0.0029; week 2 *p* = 0.0218; week 5 *p* < 0.0001; week 6 *p* = 0.0009), and interactive effects on three sample weeks (week 1 *p* = 0.008; week 5 *p* = 0.0004; week 6 *p* = 0.0038). Amending substrates with AA reduced phosphorus loss compared to all unamended substrates over the first two sample weeks. By the third week, or one week after the conclusion of the 2-week phosphorus fertilizer treatment, differences between treatments became more muted—as only the PL4 substrate had a greater P concentration than any of the AA-amended substrates, with all other substrate/fertilizer treatments equivalent to each other. Samples collected on week 4 had a greater concentration of P in all PL treatments than any AA treatment, regardless of fertilizer practice. Over the final two weeks, the only replicates receiving P were the PL6 and AA6 treatments. During these two sample weeks, PL6 had a greater P concentration in leachate than any other treatment.

**Figure 3:**
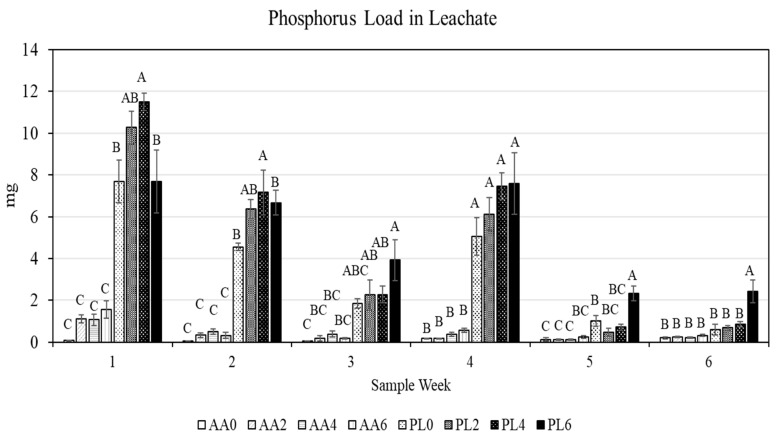
Phosphorus load in container leachate throughout the study (mean and standard error displayed; Tukey’s honestly significant difference at α = 0.05 on a given date represented by varying letters). Treatments include substrate (PL = unamended peat substrate; AA = activated aluminum-amended peat substrate) and weeks receiving phosphorus fertilizer (0, 2, 4, or 6 weeks).

The load of P collected in weekly samples followed similar patterns as the concentration of P, as the volumes of leachate were effectively equivalent (Figure 3). With leachate volumes oftentimes under 1 L, the load of P collected (in mg) was lower than the concentration (mg L^−1^) in several cases. Substrate type had a significant main effect on P load for all six sample weeks (*p* < 0.0001 for every week), fertilizer practice for 4/6 weeks (week 1 *p* = 0.0161; week 2 *p* = 0.02; week 5 *p* < 0.0001; week 6 *p* = 0.0006), and an interactive effect for 3/6 weeks (week 1 *p* = 0.0418; week 5 *p* = 0.0004; week 6 *p* = 0.002). Over the first two sample weeks, all PL treatments leached a greater load of P than all AA treatments. Similarly, in week 4, all PL treatments leached more P than all AA treatments. Weeks 3, 5, and 6 reflected some differences between treatments; however, these were largely localized to PL6 being greater than all AA treatments (and some PL treatments in weeks 5 and 6).

In assessing the cumulative load of P leached from containers throughout the entire study, AA-amended substrates reduced P load leached compared to all PL treatments, regardless of fertilizer practice (Table 1). The total amount of P recovered in container leachate was greatest in PL2, PL4, and PL6, whereas PL0, which did not receive any exogenous P beyond the starter charge, leached more P than any AA treatment and less P than any other PL treatment. In assessing cumulative P leachate collected, substrate (*p* < 0.0001) and fertilizer practice (*p* < 0.0001) each had a main effect as well as an interactive effect (*p* = 0.0034).

#### 2.2.4. Potassium Leachate

Potassium lost to leachate was equivalent between all substrate treatments and fertilizer practices on each sample day, with regards to both concentration and load (Table 1). While the concentration of K lost to container leachate was equivalent between treatments on all days, there was a significant main effect for substrate on weeks 2 and 6. Similarly, no differences were observed between treatments in the load of K recovered; however, a significant substrate main effect was identified in weeks 2 (*p* = 0.0116) and 6 (*p* = 0.0059). In assessing the cumulative load of K recovered throughout the study, there were no differences between treatments; however, the substrate main effect was significant (*p* = 0.0094).

#### 2.2.5. Micronutrients (Calcium, Chlorine, Iron, Magnesium, Manganese, Sodium, and Sulfur)

Several micronutrients and other ions of interest were investigated in this study, with the total load of each ion collected throughout the entire study presented in Table 1. Calcium, chlorine, iron, magnesium, manganese, sodium, and sulfur content in container leachate were all assessed, with generally no differences between substate treatments and fertilizer practices.

Calcium concentrations were effectively equivalent, save for minor differences in the first sample week, when PL4 was greater than PL6. There were no main effects (*p* > 0.05) for substrate type or fertilizer practice on any sample date when assessing concentration of calcium in container leachate, nor were there any interactive effects. Similarly, the load of calcium recovered from container leachate was equivalent between treatments on all sample days following week 1, although a significant substrate main effect (*p* = 0.0082) was observed during that initial week. The total amount of calcium collected was equivalent between all treatments, with no main effects exhibited for either substrate type (*p* = 0.2083) or fertilizer practice (*p* = 0.3897), nor any interactive effects (*p* = 0.5335).

Chlorine concentrations were equivalent between treatments for all six sample weeks; however, a main effect on substrate was significant in weeks 2 (*p* = 0.0023), 4 (*p* = 0.0025), and 6 (*p* = 0.0013). The load of chlorine recovered from leachate exhibited minor differences between treatments in weeks 1 and 2, but from week 3 forward there were no differences between treatments. There were significant main effects for substrate type on chlorine load lost to leachate in week 2 (*p* = 0.0063), and fertilizer practice in weeks 1 (*p* = 0.0179) and 2 (*p* = 0.0417). The total load of chlorine recovered was equivalent between all treatments; however, a main effect for substrate type was significant (*p* = 0.0208).

In assessing iron, a substrate main effect (*p* < 0.0033) was significant for all six sample dates when assessing concentration, and 5/6 sample dates when assessing load. There were no fertilizer practice main effects, nor interactive effects between substrate and fertilizer on any individual sample date (*p* > 0.05). The total load of iron was generally equivalent between all treatments; however, PL0 was greater than all AA treatments. Similarly, a substrate main effect (*p* < 0.0001) was observed in assessing cumulative iron load collected. Iron concentrations in container leachate were universally low, never exceeding 0.37 mg L^−1^.

Magnesium was generally equivalent between all treatments, where all treatments had equivalent concentrations collected in leachate for all sample weeks after week 1. There were no main effects or interactive effects from substrate and fertilizer practice on magnesium concentration on any sample date (*p* > 0.05). For the load of magnesium collected in container leachate, there were no differences between treatments on any individual sample date, nor for cumulative magnesium collected. Despite no differences between treatments in the load of magnesium recovered, there was a significant (*p* = 0.0237) main effect for substrate in week 1.

Manganese concentration and load were equivalent between all treatment combinations for each sample date, and in assessing cumulative manganese recovered in container leachate. Despite no differences in concentration or load of manganese recovered in container leachate between treatments, a significant main effect for fertilizer practice was observed in week 4 for concentration (*p* = 0.048) and load (*p* = 0.0475), and in assessing cumulative manganese load recovered (*p* = 0.0442).

There were differences in sodium concentration between treatments in weeks 1, 2, 4, and 6, generally with PL leaching slightly less sodium than AA-amended substrates. In assessing sodium concentration, a substrate main effect was significant on 5/6 sample dates with no fertilizer practice main effects or interactive effects significant on any date. Despite differences in the concentration of sodium in container leachate, the load recovered was generally equivalent for each sample week except for week 5. In assessing total sodium recovered from container leachate, AA4 was greater than PL0 and PL2; however, there were no differences between treatments in any other regard, and only a significant substrate main effect was observed (*p* = 0.0004).

Sulfur concentration and loads were equivalent between treatments on all sample dates. For both the concentration and load of sulfur in container leachate, a substrate main effect was significant during weeks 2 (*p* = 0.0023), 4 (*p* = 0.0025), and 6 (*p* = 0.0013). There were no fertilizer main effects or interactive effects between substrate and fertilizer for either concentration or load on any sample date throughout the study. There were no differences between treatments in the total cumulative load of sulfur recovered in container leachate; however, there was a strong (*p* < 0.0001) main effect for substrate.

## 3. Discussion

Short-cycle floricultural crops require readily available nutrients to sustain growth, with fertigation providing direct feeding to plant roots [34,35]; however, efforts to limit the loss of essential elements can be achieved by (1) increasing substrate nutrient retention capacity through amendments that alter substrate charge state [16] and (2) reducing superfluous nutrient applications [36]. Barriers to implementation in floricultural production include the wide range of taxa produced, as reduced nutrients and potential phytotoxic effects of Al may prove deleterious to crops. In the previous iteration of this research [17], Al leaching was equivalent (and in some cases, reduced) when substrates were amended with AA and no deleterious effects on *Tagetes* growth were observed; therefore, Al content in leachate was not quantified in this study. Similarly to previous reporting by Abdi et al. [17], the growth of *Tagetes* was not affected by the presence of AA, alleviating concerns regarding phytotoxicity to this particular taxa. Maintaining a suitable pH for AA stability [33] and nutrient availability for crops [32] may serve as an indicator of the relative risk of Al toxicity or leaching loss. As both leachate pH and crop size and quality in this study were generally equivalent, concerns over nutrient availability and potential phytotoxic reactions from AA were not realized.

Indeed, amending substrates with AA and reducing P applications were individually effective means to limit P loss to the environment without sacrificing crop quality; however, incorporating AA into substrates may be a preferred practice in short-cycle floriculture production. It was observed that in PL0 replicates (where no exogenous P was supplied), the concentration of P in leachate was similar to treatments receiving exogenous P, indicating a starter-charge of P fertilizer already present in the PL mix. Similar concentrations of P between PL0 and PL2, PL4, and PL6 were maintained over the first four weeks of the study, before the initial P in the PL substrate may have been exhausted (either via plant uptake or leachate loss). As equivalent growth indices and shoot biomass of *Tagetes* were achieved for all treatment combinations, this suggests that the initial starter-charge of P present in the commercial blend was sufficient to achieve desirable growth in *Tagetes*, and that P supplied in excess may not enhance crop quality and would contribute to increased environmental loss. Within PL replicates, P leachate losses were reduced by 32.2, 14.6, and 2.2% when P was supplied for 0, 2, and 4 weeks, respectively, compared to the entire 6-week production period. This suggests that developing improved retention of P may be preferable towards modifying P application practices, particularly when P is already present in commercially available substrate blends.

The retention of P when grown in AA-amended substrates ranged from 89.5–97.7% when compared to PL substrates receiving P the entire 6-week production period. This highlights the capacity for AA to maintain P-sorption efficacy without sorption-site saturation, at least in short-term production cycles (~6 weeks as herein). In comparing between PL0 (no AA to sorb P; no P addition beyond PL starter charge) and AA6 (AA incorporated to sorb P and the greatest amount of P added), nearly 6.5 times as much P leached from PL0 as AA6. This indicates that using AA as a substrate amendment may mitigate the environmental impact of overapplying P, leaving the economic incentive to reduce P inputs as the primary consideration. The benefits of using aluminum materials to manage P may afford other opportunities in plant production, like serving as a source of P. Research by Lin et al. [37] investigated P management using alumina materials in the production of *Tagetes* from a different approach—using P-enriched alumina as a substrate amendment to both supply P to plants and sequester P from leaching. The results of their experiments indicated that even their lowest rate of P-enriched alumina (1% by volume) supplied sufficient P for crop nutrition (as plants were of equivalent height regardless of P treatment), and that P leaching losses were substantially reduced when P-enriched alumina was added to the substrate. The similarities between Lin et al.’s [37] research and this project reflect *Tagetes*’ robustness to a range of P application rates and sources and the capacity for aluminum products to limit P loss. Furthermore, the majority of P leaching from all substrates in both studies occurred over the first three weeks of a 6-week (this study) and 8-week [37] study. A study by Borch et al. [38] identified other benefits that alumina-amended substrates may provide in the production of *Tagetes patula*, such as improved drought tolerance and root architecture (a longer primary root and enhanced lateral root distribution throughout the substrate). Spanning several studies investigating aluminum products effects on the growth of *Tagetes patula* specifically, incorporation of aluminum at low rates can effectively retain P regardless of whether it is enriched with P before blending [37] or not [17] and may confer further benefits in terms of drought tolerance and influencing root morphology [38].

While the addition of AA to the substrate substantially modified P leaching, other ions of interest were largely unaffected. With regards to micronutrient leaching, total losses of Ca, Mg, Mn, Cl, K, and S were universally equivalent between all substrate and P fertilizer treatment combinations. Only Na and Fe exhibited any differences in total leaching losses, with the former slightly greater in AA leachate and the latter slightly greater in PL leachate. This demonstrates the specificity with which AA may control for leaching of P without modifying the behaviors of other necessary ions.

To expand the widespread implementation of AA as a substrate amendment in short-cycle crops, a greater variety of floricultural taxa must be assessed in order to dissuade concerns over Al toxicity. Furthermore, the use of AA as a substrate amendment for plants grown in longer production cycles, such as nursery crops, would similarly need to demonstrate resilience to any potential phytotoxic effects of Al and maintain P sorption capacity over a longer duration. Finally, AA use as a substrate amendment must be economically feasible. Based on AA pricing information provided by the manufacturer and the rate of AA blended into media in this study, it would cost approximately $27.99 USD to amend one m^3^ of substrate with AA. This would translate to approximately $0.08 USD per 1 gallon container. Mixing AA into substrates may not necessarily incur additional equipment costs, as many growers will already have the infrastructure to readily amend substrates on-site. Growers must weigh the financial cost of implementation (AA material) to the cost of P lost to leachate and subsequent downstream release (which bears economic, ecologic, and potentially legislative implications).

## 4. Materials and Methods

### 4.1. Substrates and Leachate Collection

All substrates were comprised of a commercially available Canadian sphagnum peat moss-based mix with a starter fertilizer charge (PRO-LINE C/P Growing Mix, Oldcastle Lawn & Garden, Atlanta, GA, USA) amended with 0.6 kg per m^3^ of a micronutrient blend (Micromax SKU G90505, ICL Fertilizer, Dublin, OH, USA). The control substrate (PL) had no AA added, while the AA treatment (AA) was the same substrate amended with AA at a rate of 12.7 kg m^3^ (Riverland Industries, Baton Rouge, LA, USA). Thirty-two containers (Pro Cal 1 Gal HGPK1PHD, Southgate, CA, USA) were filled with either substrate (16 with AA (AA) and 16 without AA (PL)) on 20 July 2023. Containers filled with substrate were placed in a secondary dedicated container (2.37 L Measure-Right Mixing Container Model # PN0032; United Solutions, Leominster, MA, USA) to collect all container leachate. Supporting the plant container was a plastic cup (6.5 cm bottom diameter, 10 cm top diameter, 11.5 cm height), with a slit down the side to provide sufficient height over the container leachate and prevent substrate reabsorption.

### 4.2. Plants

French marigold (*Tagetes patula*) seeds (‘Marigold French Bonanza Yellow’ ‘PAS2276’ PanAmerican Seed, West Chicago, IL, USA) were planted in trays on 26 June 2023 and maintained in the HRS headhouse until transplanting into the trade 1 gallon container substrates on 20 July 2023.

### 4.3. Irrigation

Irrigation was applied via individual container spray stakes (Spray Stake: Model #22500-002030; Drip Tube Assembly Model #40201-002020, Netafim, Tel Aviv, Israel), providing 12.1 L h^−1^. Irrigation was activated daily at 10:00 A.M. via a battery-operated hose-end timer (DIG BO9D Hose-End Timer, Vista, CA, USA), applying 200 mL (1 min) each day throughout the study, with minor exceptions. Irrigation was increased to two minutes per day (400 mL) between 18 August and 24 August to accommodate increased temperatures. On sample collection days, the irrigation timer was deactivated to allow for leachate collection from the previous week and to apply fertigation, and then reactivated to apply irrigation immediately following fertigation. At the conclusion of the first fertigation event, 2 min of irrigation was applied; however, during all subsequent fertigation events, only 1 min of irrigation was applied.

### 4.4. Fertigation

Individual replicates were hand-fertigated weekly (following leachate collection from the week prior) with 100 mL of one of two fertilizer solutions (a solution containing P, and a solution excluding P). Solutions contained 6.66 g Ammonium sulfate (Hi Yield Ammonium Sulfate (21-0-0), product #32177, Bonham, TX, USA), 3.11 g Super Phosphate (Hi-Yield Triple Super Phosphate (0-0-60), product #32275, Bonham, TX, USA), and 2.33 g Potash (Hi-Yield Muriate of Potash (0-0-60), product #32145, Bonham, TX, USA) to provide 200 mg L^−1^, respectively, when mixed with 7 L of distilled water; with the second fertilizer solution the exact same, but without phosphate. Fertigation scheduling for plants followed a 0-, 2-, 4-, or 6-week schedule for receiving phosphate, where 0 weeks indicates never receiving fertigated phosphate, and 2, 4, and 6 indicating the number of weeks replicates received fertigated phosphate throughout the duration of the six-week study. Following fertigation, replicates received their daily irrigation volume. Within this study, PL0 represents the leaching dynamics of the starter charge of P, with no additional P added nor AA to sequester the initial P.

### 4.5. Sample Collection

Leachate samples were collected six times throughout the study: on 4, 11, 18, and 25 August, and 1 and 8, September, respectively representing weeks 1–6 of this study. On sample collection days, the automated irrigation was deactivated, and water volumes were measured within collection containers for pH and EC using a meter (GroLine Model #HI9814, Hanna Instruments, Woonsocket, RI, USA). Leachate volumes were measured with graduated cylinders, with 40 mL subsamples collected in glass amber bottles (Product #B76840—40 mL Amber VOA vials, Scientific Specialties, Hanover, MD, USA) for laboratory analysis. Samples were kept refrigerated until analysis at the Louisiana State University Soil Testing and Plant Analysis Laboratory (Louisiana State University, Baton Rouge, LA, USA), and analyzed for Ca, Cl, Fe, K, Mg, Mn, Na, P, and S using inductively coupled plasma (ICP) optical emission spectroscopy (ICP SPECTRO ACRCOS Model FHE12, Kleve, Germany). Following each leachate collection date, collection buckets were triple rinsed with distilled water.

### 4.6. Growth and Quality of Marigolds

Growth index was measured on 11 August (week 2), 25 August (week 4), and 8 September (week 6). Plant quality ratings were subjectively rated on a scale of 1–9 (with increasing number indicating higher quality) on 8 September. Shoots were severed at the substrate base on 8 September and oven dried at 100 °C until 11 September.

Growth Index=(Width 1×Width 2×Height)÷3

### 4.7. Experimental Design

This study employed a completely randomized design for all treatment combinations. Treatments were described as a combination of substrate (AA or PL) and weeks with P added (0, 2, 4, or 6); for example, AA0 indicates the substrate was amended with activated aluminum and received zero weeks of fertigation supplied P. In total, there were 8 treatments: AA0, AA2, AA4, AA6, PL0, PL2, PL4, and PL6. Each treatment had four replicates (n = 4) for a total of 32 experimental units, Individual container/leachate collection system were assigned numbers 1–32, with substrate type (with or without AA), and fertilizer schedule (0, 2, 4, or 6 weeks of P) randomly assigned using a random number generator.

### 4.8. Data Analysis

Data were analyzed using statistical analysis software (JMP Pro 17.0.0 Software; SAS Institute Inc., Cary, NC, USA) to assess means, standard errors, and Tukey’s Honestly Significant Difference at α = 0.05.

## 5. Conclusions

Amending floricultural substrates with AA is an effective way to reduce P leachate losses without needing to modify (i.e., reduce) P application rates; however, for floriculture crops on short production cycles, such as *Tagetes patula*, exogenous P applications may also be reduced without detriment to crop quality, especially if commercially available medias are pre-charged with nutrients. Insights from this research further emphasize the capacity for AA to limit P leaching (environmental concern), and that P management practices for short-term floriculture crops may be worth re-examining (for economical reasons). This research identifies two management considerations (substrate management and fertilizer management) for which floricultural growers may use to improve short-cycle crop production, alone or in tandem. However, widespread implementation of AA as a substrate amendment demands expanded knowledge on potential Al sensitivity of a wider range of floricultural and nursery crops to employ this practice en masse throughout a greenhouse or nursery operation.

## Figures and Tables

**Table 1:** Cumulative load of elements recovered from container leachate over the six-week study period (mean and standard error displayed; Tukey’s honestly significant difference at α = 0.05 between treatments represented by varying letters). Treatments include substrate (PL = unamended peat substrate; AA = activated aluminum-amended peat substrate) and weeks receiving phosphorus fertilizer (0, 2, 4, or 6 weeks).

	Total Load (mg) of Elements Recovered in Leachate over Six Weeks							
Substrate/Fertilizer	AA0	AA2	AA4	AA6	PL0	PL2	PL4	PL6
Element:								
Calcium	94.4 (±14.5)	84.4 (±7.57)	89.7 (±12.1)	86.3 (±5.42)	103.3 (±5.83)	87.7 (±5.29)	87.2 (±10.2)	109.6 (±6.22)
Chlorine	113.9 (±8.61)	110.6 (±6.44)	112.6 (±3.44)	107.1 (±5.36)	112.9 (±2.50)	97.8 (±2.59)	95.2 (±4.31)	102.5 (±4.63)
Iron	0.66 (±0.08) C	0.63 (±0.04) C	0.66 (±0.07) BC	0.68 (±0.04) BC	1.11 (±0.06) A	0.93 (±0.04) AB	0.88 (±0.06) ABC	0.89 (±0.06) ABC
Magnesium	32.5 (±4.95)	30.8 (±2.02)	29.0 (±2.56)	33.2 (±2.46)	37.3 (±0.77)	33.8 (±3.08)	33.9 (±4.28)	32.4 (±1.52)
Manganese	0.015 (±0.004)	0.012 (±0.003)	0.009 (±0.001)	0.005 (±0.001)	0.014 (±0.002)	0.009 (±0.001)	0.010 (±0.004)	0.007 (±0.001)
Phosphorus	0.68 (±0.16) C	2.20 (±0.10) C	2.68 (±0.39) C	3.20 (±0.70) C	20.8 (±0.92) B	26.2 (±1.10) A	30.0 (±1.79) A	30.6 (±1.44) A
Potassium	238.4 (±32.1)	225.1 (±12.3)	212.7 (±16.2)	234.9 (±15.1)	211.8 (±6.04)	188.0 (±8.55)	194.8 (±17.5)	187.0 (±3.77)
Sodium	412.3 (±25.7) AB	429.9 (±27.4) AB	451.6 (±9.60) A	397.1 (±10.7) AB	367.2 (±11.1) B	371.7 (±9.58) B	374.3 (±17.1) AB	380.6 (±13.7) AB
Sulfur	283.0 (±39.7)	290.5 (±15.1)	285.1 (±13.8)	299.4 (±14.7)	247.1 (±4.15)	218.6 (±18.7)	213.6 (±21.3)	219.1 (±5.14)

## Data Availability

Data will be made available upon request.
